# Physical activity and its associated factors and health correlates: a cross-sectional study of the occupational population in China’s economically developed regions

**DOI:** 10.3389/fpubh.2026.1862393

**Published:** 2026-09-22

**Authors:** Biandi Li, Jiamin Jiang, Qiang Hu, Yuyang Wang, Xinqi Cai, Defu Ma

**Affiliations:** 1 Peking University Health Science Center‑Life Talk Lifestyle Medicine Joint Laboratory, Beijing, China; 2 Department of Social Medicine and Health Education, School of Public Health, Peking University Health Science Center, Beijing, China

**Keywords:** cross-sectional associations, influencing factors, multilevel analysis, occupational population, physical activity

## Abstract

**Objective:**

This study aims to investigate physical activity behavior among occupational populations in China’s economically developed regions, identifies associated factorsinfluencing factors, and explores its cross-sectional associations with multiple health indicatorshealth effects.

**Methods:**

A cross-sectional study was conducted on 6,836 employees from 64 enterprises in Beijing, Shanghai and Guangdong using 2024 Asia Best Workplace Selection Project data. Descriptive statistics, chi-square test, rank sum test and two-level logistic regression were applied to analyze activity status, influencing factors and health effects.

**Results:**

Only 37.9% of participants met sufficient activity standards. Individual factors (male gender, age ≥40 years, unmarried status, higher education, no overtime, no perceived exercise barriers) and enterprise factors (equipped fitness facilities, provided health checkups) were significantly associated with higher odds ofwere protective factors for sufficient exercise (*p* < 0.05). Insufficient activity was associated with higher prevalence of WMSDs, depression, and anxiety symptoms (*p* < 0.05), and also with a significantly different overall BMI distribution, though the combined overweight/obesity prevalence did not differ between groups. Insufficient activity was associated with higher prevalence of overweight/obesity, work-related musculoskeletal disorders (WMSDs), depression and anxiety (*p* < 0.05).

**Conclusion:**

Physical activity levels of occupational populations in China’s economically developed regions are relatively low, affected by individual and enterprise factors. Sufficient physical activity was associated with a lower prevalence of multiple physical and depressive and anxiety symptomsmental health problems, providing empirical support for formulating targeted occupational health promotion strategies.

## Introduction

In contemporary workplace settings, insufficient physical activity has emerged as a significant global public health challenge. A plethora of studies have indicated that occupational groups across the globe predominantly engage in sedentary work patterns, with very limited time devoted to moderate-to-vigorous physical activity (1). This issue is particularly pronounced in China, where only 25.7% of the vast occupational population achieves the recommended level of moderate-to-vigorous physical activity (2). This figure is substantially below the 40% target set by the “Healthy China 2030” initiative (3), and there are significant disparities in physical activity patterns exist across age and gender groups (4).

As a crucial means of maintaining and promoting health, physical activity plays an irreplaceable role in the physical and mental well-being of occupational populations. Regular physical activity has been demonstrated to reduce the risk of chronic diseases such as obesity, cardiovascular diseases, and metabolic syndrome among working adults (5–7), alleviate job burnout and work-related stress, improve occupational well-being, and reduce symptoms of anxiety and depression (8, 9). In addition, physical activity contributes to better sleep quality, mitigation of musculoskeletal disorders, and enhancement of overall quality of life (10–14).

Researchers worldwide have explored the influencing factors of physical activity among occupational groups using various behavioral theories. It is widely recognized that multiple dimensions, including individual characteristics, environmental contexts, work-related factors, and psychological perceptions, collectively shape physical activity behaviors (15, 16). Beyond these individual-level determinants, emerging evidence highlights the organizational work environment—such as job demands, support, and resources—as a key area of work life that significantly influences employee health behaviors and outcomes (17). Despite extensive research evidence on physical activity behaviors among specific populations such as adolescents and the older adults, studies targeting China’s working population remain relatively scarce (18). Existing research predominantly focuses on single organizations or specific industries, with insufficient sample heterogeneity, thereby limiting the generalizability of conclusions (19). Concurrently, mainstream analytical approaches rely on single-level models at the individual level, overlooking employees’ clustering within organizations and their cross-level interactions with occupational-environmental characteristics (20). This may underestimate group effects and overemphasize individual explanatory power.

To address these gaps, this study leverages a large multi-province sample, employs multilevel modeling to account for the nested data structure, and examines modifiable workplace factors. We selected Beijing, Shanghai, and Guangdong as they are highly urbanized regions with diverse enterprises and pronounced work-health challenges, though findings may not generalize to less developed areas. Accordingly, this study aims to systematically assess the physical activity status of working populations across these regions and to identify key determinants at both individual and enterprise levels. We hypothesized that: (H1) individual factors (male gender, older age, higher education, no overtime, fewer barriers) would be associated with lower odds of insufficient activity; (H2) workplace provisions (fitness facilities, health checkups) would be associated with higher odds of sufficient activity; and (H3) sufficient activity would be associated with lower prevalence of overweight/obesity, WMSDs, depression, and anxiety.

## Research subjects and methods

### Study population

This cross-sectional study used data from the 2024 Asia Best Workplace Project, a survey based on voluntary enterprise participation, jointly conducted by AIA Life Insurance Company Limited and the Peking University School of Public Health. Data were collected via online questionnaires from April to June 2024.

Enterprises that registered for the project were first required to complete an enterprise-level assessment. Of the 125 enterprises initially invited, 74 completed the assessment. From these, we retained 64 enterprises located in the three purposively selected regions—Shanghai, Guangdong, and Beijing for further employee sampling. Within these regions, we applied a mixed sampling approach: (1) enterprises were stratified by industry type, enterprise size, and ownership type (state-owned, joint ventures, private, wholly foreign-owned), with random selection from each stratum; (2) employees within selected enterprises were sampled proportionally to enterprise size using company rosters as sampling frames. A total of 7,004 employees were invited. After excluding 168 invalid questionnaires (logical inconsistencies or missing data on key variables), 6,836 employees were retained, yielding a valid response rate of 97.6%. The regional allocations (Shanghai 35%, Guangdong 40%, Beijing 25%) were pre-set operational targets rather than population-weighted proportions. The sample included regular full-time and dispatched workers, excluding temporary staff and those participating in similar concurrent studies.

Sample size was calculated using the formula *n* = (*Z*^2^ × P × (1-P))/*d*^2^, where *Z* = 1.96, *p* = 0.50, and d = 0.015, yielding a minimum of 4,268 participants. Accounting for 20% non-response and a design effect of 1.6 for multilevel modelling, the target sample size was approximately 6,800. The final sample of 6,836 employees provided 80% power to detect an odds ratio of 1.20 or larger at *α* = 0.05 (21).

### Data collection and measures

Data collection was conducted via online questionnaires between April and June 2024. The questionnaire was designed by a team of experts from the School of Public Health of Peking University. The content covers socio-demographic information (including gender, age, marital status, education, occupation, etc.), behavioral and lifestyle factors (such as physical activity, smoking status, sleep quality, etc.), work-related attributes and health awareness level. All questionnaires used logical verification, missing data detection and jump logical verification procedures to ensure data integrity.

### Relevant indicators and definitions

The level of physical activity was assessed using the Chinese version of the International Physical Activity Questionnaire Short Form (IPAQ-SF) (22, 23), which has demonstrated acceptable validity and reliability in Chinese adult and occupational populations (24, 25). The questionnaire assessed three activity domains over the past 7 days: walking (e.g., walking for at least 10 min continuously), moderate-intensity activity (e.g., carrying light loads, cycling at a regular pace), and vigorous-intensity activity (e.g., heavy lifting, aerobics, fast cycling). For each domain, participants reported the frequency (days per week) and average duration (minutes per day). Metabolic equivalent (MET)-minutes per week were calculated as MET level × minutes/day × days/week, with MET assignments of 3.3 for walking, 4.0 for moderate, and 8.0 for vigorous activity. Total MET-min/week was the sum across all three domains. According to the standard IPAQ scoring protocol, activity was categorized as insufficient (<600 MET-min/week) or sufficient (≥600 MET-min/week). Height and weight were self-reported. Body Mass Index (BMI) was classified according to the Guidelines for Prevention and Control of Overweight and Obesity in Chinese Adults into four categories: underweight (BMI < 18.5 kg/m^2^), normal weight (18.5 ≤ BMI ≤ 23.9 kg/m^2^), overweight (24 ≤ BMI ≤ 27.9 kg/m^2^), and obesity (BMI ≥ 28 kg/m^2^) (26). WMSDs were assessed using a 5-item scale adapted from the Nordic Musculoskeletal Questionnaire (27). Participants reported pain or discomfort in five body regions (hands, shoulders/neck/upper back, waist, knees, and feet) over the past 12 months. For each region, responses were scored as: none = 3, mild = 2, moderate = 1, severe = 0; the total score ranged from 0 to 15, with higher scores indicating less severe musculoskeletal symptoms (i.e., better musculoskeletal health). The composite score across multiple regions was used as an indicator of overall WMSDs severity, as WMSDs commonly involve multiple anatomical sites simultaneously, and a composite measure captures the total musculoskeletal burden more comprehensively than single-site assessment. The scale demonstrated acceptable internal consistency in this study (Cronbach’s *α* = 0.766). Depressive symptoms were measured using the 9-item Chinese version of the Center for Epidemiological Studies Depression Scale (CES-D-9), with scores from 0 to 27 (cutoffs: ≥10 for mild symptoms, ≥19 for moderate-to-severe symptoms) and a Cronbach’s α of 0.863 (28). Anxiety symptoms were assessed with the Generalized Anxiety Disorder 2-item (GAD−2) scale, which has a score range of 0–6(cutoff: ≥3 for significant anxiety symptoms) and a Cronbach’s α of 0.797 (29).

### Multilevel model

Multilevel models are statistical analysis methods specifically designed for hierarchical data structures, particularly suited for non-independent data. As a type of multilevel model, multilevel logistic regression is applicable to studies using discrete variables as analytical factors (30, 31). It is appropriate for the data in this study (which exhibits a company-employee hierarchical structure), enabling the separation of variation at the company level to more accurately analyze the factors influencing physical activity among working populations. Covariates were selected based on the socio-ecological model and prior empirical evidence, encompassing both individual-level characteristics and enterprise-level environmental factors (15, 16). By including variables from both levels simultaneously in the random-intercept model, we adjusted for potential confounding within each level.

1. Variable Assignment: This study classified and transformed variables in the raw data to ensure the data met model requirements (Table 1).
2. Model construction: We constructed a zero model without covariates and a random intercept model incorporating covariates, using the adequacy of physical activity among working populations as the dependent variable. Employees represent Level 1, while enterprises represent Level 2.

**Table 1:** Basic variable information.

Variable	Assignment	Note
Level of physical activity	0 = Sufficient, 1 = Insufficient	Binary dependent variable
Gender	1 = Male, 2 = Female	Binary independent variable
Age/years	1 = 16–29, 2 = 30–39, 3 = ≥40	Ordered multi-class variables are incorporated as dummy variables
Marital status	1 = Unmarried, 2 = Married, 3 = Divorced/widowed	Ordered multi-class variables are incorporated as dummy variables
Education level	1 = High school and below, 2 = Junior college, 3 = Bachelor degree, 4 = Master degree and above	Ordered multi-class variables are incorporated as dummy variables
Working system	1 = Day shift, 2 = Non day shift	Binary independent variable
Weekly overtime days	1 = No overtime, 2 = 1–3 days, 3 = 4–6 days, 4 = Every day	Ordered multi-class variables are incorporated as dummy variables
Commute time	1 = <60 min,2 = ≥60 min	Binary independent variable
Lack of time	0 = No, 1 = Yes	Binary independent variable
Dislike sports	0 = No, 1 = Yes	Binary independent variable
Lack of venue or conditions	0 = No, 1 = Yes	Binary independent variable
Industry	1 = Real estate/Construction Industry, 2 = Service Industry, 3 = Computer/Internet Industry, 4 = Financial Industry, 5 = Fast Moving Consumer Goods/Retail Industry, 6 = Fast moving consumer goods/Retail Industry, 7 = Manufacturing industry	Ordered multi-class variables are incorporated as dummy variables
Scale of the Enterprises	1 = ≤500,2= > 500	Binary independent variable
Health checkup	0 = No, 1 = Yes	Binary independent variable
Fitness facilities or venues in the workplace	0 = No, 1 = Yes	Binary independent variable

First, construct a zero model that does not include any explanatory variables (32) (Model 1) as shown in Equations 1–3:

$$\text{logit}(\frac{P_{\mathrm{ij}}}{1-P_{\mathrm{ij}}})=\beta_{0j}+e_{\mathrm{ij}}$$ (1)

$$\beta_{0j}=\beta_{0}+u_{0j}$$ (2)

$$\begin{array}{c} u_{0j}\sim N(0,\sigma_{u0}^{2}) \end{array}$$ (3)

In the formula: $P_{\mathrm{ij}}$ represents the probability that the i-th employee in the j-th firm engages in insufficient physical activity. $\beta_{0j}$ represents the intercept of the j-th enterprise, under the zero model with no explanatory variables, it represents the logarithm of the advantage of “insufficient physical activity relative to sufficient activity” among employees within the j-th enterprise. $\beta_{0}$ represents the average intercept term for the entire sample, corresponding to the average logarithmic value of the advantage employees have in “insufficient physical activity relative to sufficient activity”across the entire sample range. $u_{0j}$ is the residual term at level 2, and its variance $\sigma_{u0}^{2}$ can be used to estimate the differences between enterprises.

Second, construct a random intercept model (Model 2) as shown in Equations 4, 5: Sequentially introduce the explanatory variables: age, gender, marital status, education level, working system, overtime status, commute time, perceived physical barriers, industry, company size, health checkups, and availability of fitness facilities.

$$\text{logit}(\frac{P_{\mathrm{ij}}}{1-P_{\mathrm{ij}}})=\beta_{0j}+\sum_{n=1}^{n=14}\beta_{n}X_{\mathrm{nij}}+e_{\mathrm{ij}}$$ (4)

$$\begin{array}{c} u_{0j}\sim N(0,\sigma_{u0}^{2}) \end{array}$$ (5)

$X_{\mathrm{nij}}$ denotes the j-th enterprise’s i-th employee’s n-th explanatory variable affecting physical activity. $\beta_{1}$ to $\beta_{14}$ denote the fixed-effect estimates of explanatory variable $X_{\mathrm{ij}}$ . $\sigma_{u0}^{2}$ represents the variance component at level 2.

### Quality control

All investigators were required to complete project-specific training and pass a qualification assessment. Data were cleaned and verified for logical consistency in accordance with relevant protocols, with all processing steps fully documented for audit purposes. The online survey required mandatory responses for core items to minimize missing data. During cleaning, we additionally excluded cases with illogical values or missing data on key variables (physical activity, BMI, WMSDs, depression, anxiety) using listwise deletion; thus, the final analytical dataset contained no missing values for the variables included in the models. Appropriate statistical methods were selected based on data characteristics and research objectives, with reference to existing literature, to ensure the reliability of the analytical results.

### Statistical analysis

Statistical analysis was performed using R4.4.3 Software. Categorical data are presented as frequencies and percentages (%), and group comparisons were made using Pearson’s chi-squared test. Continuous variables are summarized as median with interquartile range (IQR), and the Wilcoxon rank-sum test was employed for group comparisons. A two-level logistic regression model was constructed to analyze the factors associated with physical activity in the occupational population. All tests were two-sided, with a significance level set at 0.05.

### Ethical approval

This study was approved by the Institutional Review Board of Peking University Health Science Center (IRB00001052-24156). All participants provided written informed consent before completing the online anonymous questionnaire, and all research procedures were conducted in accordance with the Declaration of Helsinki (2013 revision).

## Results and analysis

### Basic characteristics of the research subjects

A total of 6,836 occupational participants were enrolled in this study, among whom 4,246 (62.1%) had insufficient physical activity and 2,590 (37.9%) met the sufficient physical activity criteria. The majority of participants were aged 30–39 years (45.2%), married (62.7%), with a bachelor’s degree (53.0%), and engaged in day-shift work (86.6%). In addition, 47.5% of the participants reported a lack of time as a barrier to physical activity, while 78.2% indicated a preference for sports. The 64 participating enterprises were located in Beijing, Shanghai and Guangdong, covering seven major industries, with the manufacturing industry accounting for the largest proportion (32.4%). Large enterprises with more than 500 employees made up 68.3% of the total. Moreover, 85.8% of the enterprises were equipped with fitness facilities in the workplace, and 78.4% of the participants had received health check-ups provided by their enterprises (Table S1).

### Univariate analysis of factors influencing physical activity among occupational populations

The univariate analysis indicated statistically significant differences in physical activity levels among employees based on gender, age, education level, working system, weekly overtime days, commute time, industry, and enterprise size (*p* < 0.05). Employees who perceived activity barriers (e.g., lack of time, dislike of sports, or lack of venues) and those whose enterprises did not provide fitness facilities or health checkups showed higher rates of insufficient activity (*p* < 0.05) (Table 2).

**Table 2:** Univariate analysis of categorical variables in the influencing factors on physical activity among occupational populations.

Variable	*χ* ^2^	*p*-value
Gender	11.260	<0.001***
Age/years	37.021	<0.001***
Marital status	2.421	0.298
Education level	124.838	<0.001***
Working system	57.620	<0.001***
Weekly overtime days	200.979	<0.001***
Commute time	7.115	0.008*
Lack of time	435.573	<0.001***
Dislike sports	235.663	<0.001***
Lack of venues or conditions	37.820	<0.001***
Industry	57.324	<0.001***
Enterprise size	14.371	<0.001***
Fitness facilities or venues in the workplace	173.763	<0.0.001***
Health checkup	82.511	<0.0.001***

Chi-square tests were used to compare the differences in physical activity levels among occupational populations with different characteristics; * = *p* < 0.05, ** = *p* < 0.01, *** = *p* < 0.001.

### Results of the multilevel model

#### Results of the null model

This study constructed a two-level zero model without explanatory variables to examine the clustering effect of employee mobility behavior at the firm level. Results indicated significant inter-firm variation ( $\sigma_{u0}^{2}$ = 0.584, *p* < 0.05, Wald test), with ICC = 0.151, suggesting 15.1% of total variance originates from the firm level. The likelihood ratio test confirmed superior fit of the two-level model over single-level logistic regression(*p* < 0.05), supporting the adoption of a multilevel model (Table 3).

**Table 3:** Likelihood ratio test of single level null model and two-level null model.

Model	Number of parameters	*AIC*	*BIC*	*-2 Log Likelihood*	*χ^2^*	*P*
Single level null model	1	9073.529	9080.359	9071.529	581.150	<0.001
Two-level null model	2	8494.379	8508.039	8490.379		

AIC: Akaike information criterion; BIC: Bayesian information criterion.

#### Results of the two-level logistic regression

Based on the zero model confirming significant differences between groups, this study estimated a two-level logistic regression model incorporating explanatory variables. Multicollinearity diagnostics indicated no significant issues (VIFs: 1.12–2.41, all <5).

##### Influencing factors at the employee level

The results (Table 4) showed that at the individual level, age, gender, marital status, education level, weekly overtime days, and perceived barriers were all significant factors influencing employees’ physical activity. Younger employees aged 16–29 and 30–39 had approximately 1.5-fold and 1.4-fold higher odds of insufficient activity, respectively, compared with those aged ≥40 (OR = 1.530, 95% CI: 1.253–1.869; OR = 1.398, 95% CI: 1.214–1.609). Female employees had about 1.2-fold higher odds than males (OR = 1.217, 95% CI: 1.082–1.369), and married employees showed a similar elevation in risk relative to unmarried individuals (OR = 1.280, 95% CI: 1.099–1.492). A clear educational gradient emerged: compared with those holding a master’s degree or above, employees with a bachelor’s degree, junior college diploma, or high school education or below had progressively higher odds of insufficient activity, with ORs of 1.312 (95% CI: 1.123–1.532), 1.666 (95% CI: 1.351–2.057), and 2.004 (95% CI: 1.555–2.580), respectively.

**Table 4:** Two-level logistic regression analysis of factors influencing physical activity among occupational populations.

Variable	OR (95% CI)	*P*-value
Fixed effects (Intercept)	0.115 (0.065–0.202)	<0.001***
Level 1		
Age/years (≥40Ref)		
16–29	1.530 (1.253–1.869)	<0.001***
30–39	1.398 (1.214–1.609)	<0.001***
Gender (Male Ref)		
Female	1.217 (1.082–1.369)	0.001**
Marital status (Unmarried Ref)		
Married	1.280 (1.099–1.492)	0.002**
Divorced/widowed	1.413 (0.968–2.061)	0.073
Education level (Master degree and above Ref)		
High school and below	2.004 (1.556–2.580)	<0.001***
Junior college	1.666 (1.351–2.057)	<0.001***
Bachelor degree	1.312 (1.123–1.532)	<0.001***
Working system		
Day shift (Ref)	—	—
Non-day shift	1.166 (0.96–1.417)	0.122
Weekly overtime days No overtime (Ref)		
1–3 days	1.431 (1.259–1.623)	<0.001***
4–6 days	1.559 (1.248–1.948)	<0.001***
Every day	2.439 (1.833–3.246)	<0.001***
Commute time<60 min (Ref)		
≥60 min	1.159 (0.999–1.343)	0.051
Lack of time (No Ref)		
Yes	2.810 (2.498–3.16)	<0.001***
Dislike sports (No Ref)		
Yes	3.762 (3.222–4.388)	<0.001***
Lack of venues or conditions (No Ref)		
Yes	1.348 (1.185–1.534)	<0.001***
Level 2		
Industry Pharmaceutical (Ref)		
Real estate/construction	1.230 (0.626–2.415)	0.548
Service	1.508 (0.864–2.633)	0.149
Computer/internet	1.194 (0.684–2.084)	0.533
Finance	0.972 (0.529–1.786)	0.927
FMCG/retail	1.137 (0.601–2.153)	0.692
Manufacturing	1.429 (0.871–2.345)	0.157
Company size≤500 (Ref)		
>500	1.101 (0.805–1.505)	0.549
Health check Yes (Ref)		
No	1.371 (1.174–1.603)	<0.001***
Fitness facilities or venues in the workplace Yes (Ref)		
No	1.617 (1.350–1.937)	<0.001***
Random effects		
Variance of residual at level 2		<0.001***

FMCG: fast moving consumer goods; * = *p* < 0.05, ** = *p* < 0.01, *** = *p* < 0.001.

Moreover, overtime work exhibited a graded association with insufficient activity: compared with no overtime, employees working 1–3, 4–6, and 7 days of overtime per week showed progressively higher odds, with ORs increasing from 1.431 (95% CI: 1.259–1.623) to 1.559 (95% CI: 1.248–1.948) and 2.439 (95% CI: 1.833–3.246), respectively—a more than twofold difference in risk for daily overtime versus none.

Perceived barriers were among the strongest individual-level correlates: employees citing lack of time had nearly three-fold higher odds of insufficient activity (OR = 2.810, 95% CI: 2.498–3.160), while those who disliked sports showed an even larger elevation, with nearly four-fold higher odds (OR = 3.762, 95% CI: 3.222–4.388). Lack of venues or conditions also conferred a modest but significant increase in risk (OR = 1.348, 95% CI: 1.185–1.534).

##### Influencing factors at the enterprise levels

Given the cross-sectional nature of this study, all findings reported in this section reflect associations rather than causal relationships. This study also analyzed the influence of enterprise-level factors on employees’ physical activity. The results indicated that industry and company size did not show statistically significant effects on employees’ risk of insufficient activity (*p* > 0.05). However, employees who did not receive a health check had a higher risk of insufficient activity (OR = 1.371, 95% CI = 1.174–1.603). Meanwhile, the absence of fitness facilities or venues in the workplace was a significant risk factor for insufficient activity (OR = 1.617, 95% CI = 1.350–1.937).

### Cross-sectional associations between physical activity and health indicators in the occupational population

This study analyzed the association between physical activity levels and health outcomes among the occupational population. A significant association was found between physical activity levels and BMI classifications (χ^2^ = 19.314, *p* < 0.05). Further analysis with the Bonferroni correction demonstrated that compared to the physically active group, the physically inactive group exhibited a significantly higher prevalence of both underweight and obesity (*p* < 0.0125), while no significant differences were observed in the normal weight and overweight categories (*p* > 0.0125). Physical activity level was also significantly associated with both depressive status (*χ*^2^ = 114.952, *p* < 0.05) and anxiety status (*χ*^2^ = 37.901, *p* < 0.05) (Table 5).

**Table 5:** Results of *χ*² test on physical activity level and BMI classifications, depression, and anxiety among the occupational population.

Variables	Total (*n*/%)	Level of physical exercise		*χ* ^2^	*P*-value
		Insufficient (*n*/%)	Sufficient (*n*/%)		
BMI classifications					
Underweight	523 (7.65)	357 (8.41)	166 (6.41)	19.314	<0.001***
Normal weight	4,053 (59.29)	2,484 (58.50)	1,569 (60.58)		
Overweight	1744 (25.51)	1,054 (24.82)	690 (26.64)		
Obesity	516 (7.55)	351 (8.27)	165 (6.37)		
Depression status					
No depressive tendencies	5,757 (84.22)	3,421 (80.57)	2,336 (90.19)	114.952	<0.001***
Mild depressive tendencies	831 (12.16)	624 (14.70)	207 (7.99)		
High risk of depression	248 (3.63)	201 (4.73)	47 (1.82)		
Anxiety status					
No significant anxiety symptoms	6,390 (93.48)	3,908 (92.04)	2,482 (95.83)		
Presence of significant anxiety symptoms	446 (6.52)	338 (7.96)	108 (4.17)		
Total	6,836 (100)	4,246 (62.11)	2,590 (37.89)		

Chi-squared tests were used to analyze the associations between physical activity level and different health outcomes (including BMI classifications, depression status, and anxiety status) among the occupational population;*** = *p* < 0.001.

The study also examined the prevalence of work-related musculoskeletal disorders (WMSDs) among the occupational population, involving five body parts: hands, shoulders/neck/upper back, waist, knees, and feet, and analyzed the association with physical activity levels. Activity The results showed that individuals with sufficient physical activity had a higher proportion of “no pain” in all body parts compared to those with insufficient physical activity. In terms of overall severity, after quantifying WMSDs using the reverse scoring method, the median score of the sufficient physical activity group was higher than that of the insufficient physical activity group, and the difference between the two groups was statistically significant (Wilcoxon W statistic = 4,403,468, *p* < 0.05) (Figure 1 and Figure S1). This statistical difference suggests that sufficient physical activity was associated with lower severity of musculoskeletal disorders.

**Figure 1:**
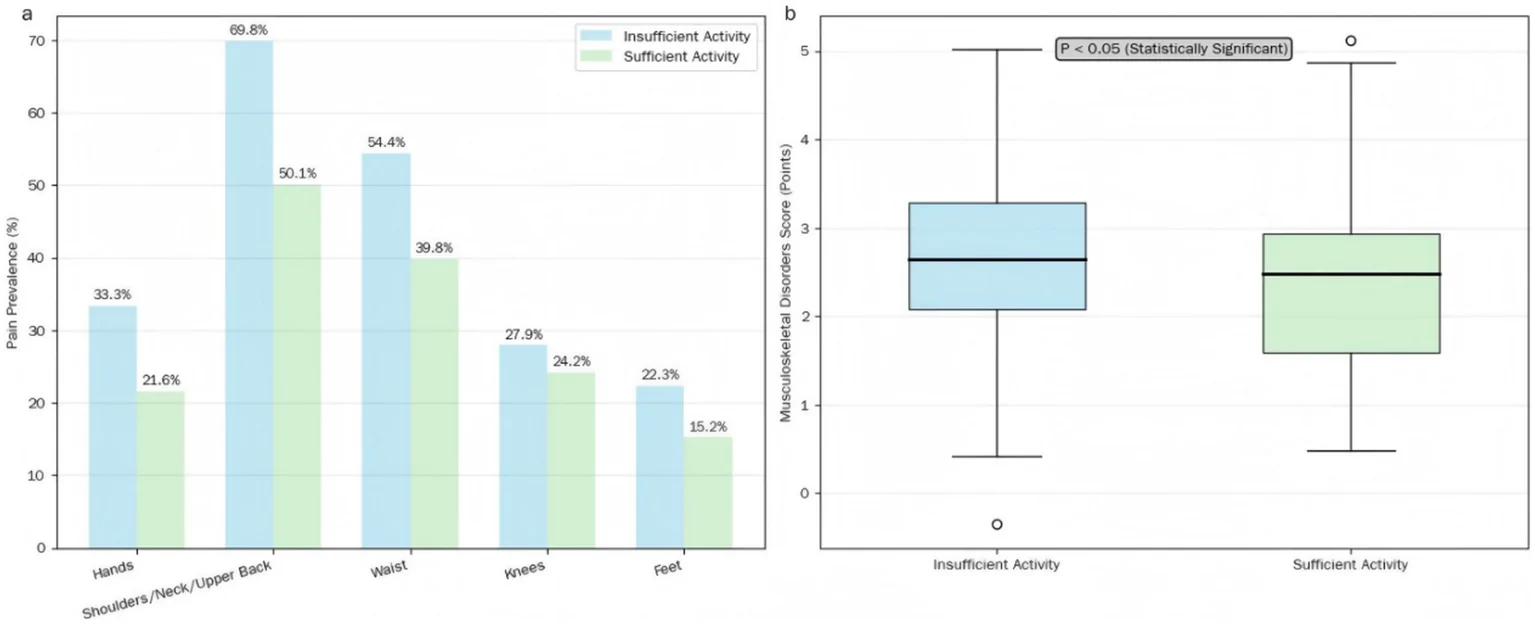
Comparison of pain distribution in various body parts and overall severity of work-related musculoskeletal disorders (WMSDs) among occupational population by physical activity level. **(a)** Pain prevalence across different body parts by physical activity level. **(b)** Quantitative comparison of overall WMSDs severity by physical activity level.

## Discussion

Based on the survey data of mainland China from the 2024 Asia Best Workplace selection project, this study examined the current status, associated factors, and cross-sectional health correlates of physical activity among 6,836 employees from 64 enterprises in Shanghai, Guangdong, and Beijing. The results showed that only 37.9% of employees met the standard of sufficient physical activity. This proportion is close to the 37.2% physical activity level of Chinese adults in the “2020 National Survey on National Fitness Activities”, higher than 15.6–34.2% in other domestic studies (2, 4, 20, 33), but remained below the Healthy China 2030 target of 40% (3). This indicates that physical inactivity constitutes a non-negligible public health concern among the occupational population in China. Consistent with our hypotheses, multilevel analysis further revealed that this behavior is jointly associated with individual factors (gender, age, education level, overtime work, perceived activity barriers) and enterprise-level factors (provision of fitness facilities and regular health checkups). Moreover, sufficient activity was associated with favorable health indicators, including lower rates of overweight/obesity, musculoskeletal disorders, and mental health issues.

Using a two-level logistic regression model, this study identified that employee activity behavior is influenced by multiple factors. At the individual level, logistic regression analysis revealed that female employees were associated with higher odds of insufficient physical activity compared to males, consistent with WHO reports (global insufficiency rates: 31.7% in females, 23.4% in males) (34),this pattern may reflect women’s dual burden of household chores and childcare, leading to compressed activity time (15, 35). Employees aged 40 and above exhibited lower risks of insufficient activity, aligning with findings from Chai Jingxin et al. (2, 36). A plausible explanation is that enhanced health awareness and reduced work pressure in this demographic, enabling better utilization of leisure time for activity (37, 38). Married employees showed higher odds of insufficient activity than unmarried individuals, consistent with research by Xiao Quanhong et al. (39, 40). This may be because unmarried individuals experienced no family role conflicts and had more free time. In terms of education level, the higher the education attainment, the lower the odds of insufficient activity, consistent with the findings of Chen Yaping et al. (41). Highly educated individuals had stronger health knowledge acquisition abilities, placed greater emphasis on activity, and possessed greater financial resources to access fitness facilities, thereby maintaining adequate activity levels (42).

The results of this study indicated that weekly overtime days were associated with physical activity levels among the occupational population. Previous studies have suggested that non-day-shift employees spend less time on physical activities compared to day-shift workers (43)., although this association did not reach statistical significance in our multivariable model. Night shifts/rotating shifts/irregular shifts may lead to circadian rhythm disruption and energy imbalance, resulting in fatigue and reduced motivation for activity (44). Employees who worked overtime in the past 6 months had higher odds of being physically inactive compared to those who did not. A study in South Korea found that as working hours increased, the participation level of employees in moderate-to-vigorous physical activity showed a declining trend (45). This may be because overtime work directly occupies the time employees could have used for physical activity. At the same time, prolonged working hours disrupt employees’ life rhythms, leaving them in a state of physical and mental fatigue, which reduces their willingness and ability to participate in physical activity.

Regarding perceived barriers, employees who mentioned obstacles such as lack of time, dislike of activity, or absence of space/conditions had a higher risk of physical inactivity compared to those without such barriers. This is consistent with previous research. Chai Jingxin et al. (36) found in their study on Beijing workers that perceived barriers limited the activity participation of the occupational population. Li Yujun et al. (19) pointed out that factors such as lack of free time, venues, adverse weather, and lack of experience collectively hindered Huaibei occupational women from engaging in activity and persisting with it.

At the enterprise level, logistic regression analysis identified the lack of workplace fitness facilities and organized health check-ups as factors significantly associated with physical inactivity among employees. Nicolaas P. Pronk (46) based on the socio-ecological model, demonstrated that workplace health promotions, organized physical activities, and the provision of fitness venues and equipment effectively encourage activity. On-site fitness facilities function as a critical environmental cue by providing convenient access, thereby facilitating regular activity and helping to establish habitual behavior. Furthermore, personalized health reports translate abstract health risks into tangible, personalized warnings, which can effectively stimulate employees’ intrinsic motivation to activity. In addition, emerging evidence suggests that workplace interventions targeting working hours and employee well-being, such as flexible scheduling and organizational support programs, may complement physical activity promotion strategies and should be considered in comprehensive workplace health initiatives (47).

Morever, this study examined associations between physical activity and multidimensional health indicators. The results showed that the overweight/obesity rate in this occupational population was 33.0%, lower than the 50.7% observed in the general Chinese population, which may be attributed to the relatively younger average age of the study participants (48). The observed cross-sectional association between sufficient activity and lower WMSDs severity is consistent with the purported biological role of physical activity in preserving motor system function (10, 49, 50). Concurrently, the association between physical activity and better depressive symptoms and anxiety symptoms underscore its psychophysiological function as an effective buffer against stress (51, 52).

Compared to previous studies on physical activity in occupational populations, this research employs a multi-level data structure and utilizes hierarchical models to analyze various associated factors, which may improve the validity and appropriateness of the interpreted results.

These findings carry several practical implications. First, at the policy level, public health strategies should prioritize support for groups at higher risk—particularly female employees, younger adults, and those with lower educational attainment—through targeted subsidies or community-based activity programs. Second, enterprises should assume core responsibility by maintaining on-site fitness facilities, offering regular health checkups with personalized feedback, and implementing flexible scheduling to reduce excessive overtime—actions directly supported by our enterprise-level findings. Third, at the individual level, employees may benefit from enhancing health literacy and utilizing fragmented time to incorporate activity into daily routines.

However, this study still has several limitations. Firstly, it is important to emphasize that due to the cross-sectional nature of this study, we cannot establish causal or temporal relationships between physical exercise and health outcomes. Reverse causality is equally plausible: individuals with existing health conditions (such as WMSDs, overweight/obesity, or mental health issues) may be less likely to engage in physical activity due to pain, discomfort, or reduced physical capacity. Secondly, all key variables including physical activity, WMSDs, depression, and anxiety were self-reported through questionnaires, which may introduce common method bias. Self-reported measures are subject to recall bias and social desirability bias, potentially leading to overestimation or underestimation of the true associations (53). Third, the WMSDs scale was adapted from the NMQ without comprehensive validation in this specific Chinese occupational population. Although the scale demonstrated acceptable internal consistency (Cronbach’s *α* = 0.766) and the NMQ has established validity internationally, construct validity and external validation against clinical assessments were not conducted. Fourth, the sampling strategy was purposive in regional selection and confined to three economically developed areas (Shanghai, Guangdong, Beijing), which limits generalizability to all of China. Moreover, enterprises enrolled voluntarily, introducing self-selection bias—a form of selection bias that limits the representativeness of our findings. We did not apply statistical weighting because our aim was descriptive within this cohort rather than inferential for the national population; weighting would not correct the inherent self-selection bias. Additionally, despite adjusting for multiple covariates, the possibility of residual confounding from unmeasured factors (e.g., health motivation, personality traits) cannot be fully excluded. Future research should employ probability-based sampling with broader geographic coverage (including central and western regions), adopt longitudinal designs, and incorporate objective measures to enhance external validity and causal inference.

In summary, current physical activity levels among Chinese employees remain suboptimal and are influenced by multifaceted factors. Addressing physical activity insufficiency within China’s working population constitutes a systematic project requiring collaborative efforts from policymakers, enterprises, and individuals. At the policy level, there should be an emphasis on strengthening guidance through forward-looking public health policies that integrate physical activity promotion into urban planning and public service systems, while providing targeted support for specific groups such as female employees and individuals with lower educational attainment. Enterprises must play a core role by optimizing work schedules, providing fitness facilities and subsidies, and organizing collective activities to foster a supportive working environment, thereby embedding health management into organizational culture. On an individual level, employees should play a principal role by enhancing health literacy, cultivating activity habits, and utilizing fragmented time effectively to internalize physical activity as a sustainable lifestyle practice.

## Conclusion

This study examined the level of physical activity among 6,836 employees from 64 enterprises in China’s economically developed regions. Only 37.9% of the working population met the recommended activity guidelines. Individual factors (gender, age, education, overtime, and perceived barriers) and enterprise factors (fitness facilities and health check-ups) significantly influenced activity behavior. Sufficient physical activity was associated with a lower risk of developing work-related musculoskeletal disorders and experiencing depressive and anxiety symptoms, and with a different overall BMI distribution pattern, but not with a lower combined prevalence of overweight/obesity. These findings provide empirical support for the formulation of targeted health promotion strategies.

## Data Availability

The datasets presented in this article are not readily available because data are subject to third-party restrictions and are not publicly available. Requests to access the datasets should be directed to BL lbiandi@163.com.
