# Supplementary material for: Physical activity and its associated factors and health correlates: a cross-sectional study of the occupational population in China’s economically developed regions
Source: Front Public Health. 2026 Sep 22;14:1862393. doi: 10.3389/fpubh.2026.1862393 (PMC13638526; doi:10.3389/fpubh.2026.1862393)
Supplement: Supplementary file 1 [file Supplementary_file_1.docx]

| **Table S1** Univariate analysis of categorical variables in the influencing factors on physical exercise among occupational populations | | | | | |
| --- | --- | --- | --- | --- | --- |
| Variable | Total (n/%) | Level of physical exercise | | χ^2^ | P-value |
|  |  | Insufficient (n/%) | Sufficient (n/%) |  |  |
| **Gender** |  |  |  |  |  |
| Male | 3054(44.68) | 1224(40.08) | 1830(59.92) | 11.260 | <0.001*** |
| Female | 3782(55.32) | 1366(36.12) | 2416(63.88) |  |  |
| **Age/years** |  |  |  |  |  |
| 16-29 | 1847(27.02) | 1194(64.65) | 653(35.35) | 37.021 | <0.001*** |
| 30-39 | 3089(45.19) | 1981(64.13) | 1108(35.87) |  |  |
| ≥40 | 1900(27.79) | 1071(56.37) | 829(43.63) |  |  |
| **Marital status** |  |  |  |  |  |
| Unmarried | 2368(34.64) | 1448(61.15) | 920(38.85) | 2.421 | 0.298 |
| Married | 4285(62.68) | 2677(62.47) | 1608(37.53) |  |  |
| Divorced/widowed | 183(2.68) | 121(66.12) | 62(33.88) |  |  |
| **Education level** |  |  |  |  |  |
| High school and below | 877(12.83) | 653(74.46) | 224(25.54) | 124.838 | <0.001*** |
| Junior college | 1048(15.33) | 718(68.51) | 330(31.49) |  |  |
| Bachelor degree | 3623(53.00) | 2193(60.53) | 1430(39.47) |  |  |
| Master degree and above | 1288(18.84) | 682(52.95) | 606(47.05) |  |  |
| **Working system** |  |  |  |  |  |
| Day shift | 5918(86.57) | 3572(60.36) | 2346(39.64) | 57.620 | <0.001*** |
| Non day shift | 918(13.43) | 674(73.42) | 244(26.58) |  |  |
| **Weekly overtime days** |  |  |  |  |  |
| No overtime | 3469(50.75) | 1893(54.57) | 1576(45.43) | 200.979 | <0.001*** |
| 1-3 days | 2386(34.90) | 1603(67.18) | 783(32.82) |  |  |
| 4-6 days | 581(8.50) | 427(73.49) | 154(26.51) |  |  |
| Every day | 400(5.85) | 323(80.75) | 77(19.25) |  |  |
| **Commute time** |  |  |  |  |  |
| <60 min | 5573(81.52) | 3420(61.37) | 2153(38.63) | 7.115 | 0.008* |
| 60 min and above | 1263(18.48) | 826(65.40) | 437(34.60) |  |  |
| **Lack of time** |  |  |  |  |  |
| Yes | 3249(47.53) | 1600(49.25) | 1649(50.75) | 435.573 | <0.001*** |
| No | 3587(52.47) | 2646(73.77) | 941(26.23) |  |  |
| **Dislike sports** |  |  |  |  |  |
| Yes | 5347(78.22) | 3067(57.36) | 2280(42.64) | 235.663 | <0.001*** |
| No | 1489(21.78) | 1179(79.18) | 310(20.82) |  |  |
| **Lack of venues or conditions** |  |  |  |  |  |
| Yes | 5062(74.05) | 3036(59.98) | 2026(40.02) | 37.820 | <0.001*** |
| No | 1774(25.95) | 1210(68.21) | 564(31.79) |  |  |
| **Industry** |  |  |  |  |  |
| Real estate/Construction Industry | 527(7.71) | 351(66.60) | 176(33.40) | 57.324 | <0.001*** |
| Service Industry | 804(11.76) | 504(62.69) | 300(37.31) |  |  |
| Computer/Internet Industry | 739(10.81) | 407(55.07) | 332(44.93) |  |  |
| Financial Industry | 1191(17.42) | 724(60.79) | 467(39.21) |  |  |
| Fast Moving Consumer Goods/Retail Industry | 433(6.33) | 262(60.51) | 171(39.49) |  |  |
| Pharmaceutical/Pharmaceutical Industry | 928(13.58) | 519(55.93) | 409(44.07) |  |  |
| Manufacturing industry | 2214(32.39) | 1479(66.80) | 735(33.20) |  |  |
| **Enterprise size** |  |  |  |  |  |
| 500 persons and below | 2165(31.7) | 1274(58.8) | 891(41.2) | 14.371 | <0.001*** |
| 500 persons and above | 4671(68.3) | 2972(63.6) | 1699(36.4) |  |  |
| **Fitness facilities or venues in the workplace** |  |  |  |  |  |
| Yes | 5866(85.81) | 3828(65.26) | 2038(34.74) | 173.763 | <0..001*** |
| No | 970(14.19) | 418(43.09) | 552(56.91) |  |  |
| **Health checkup** |  |  |  |  |  |
| No | 1478(21.62) | 1068(72.26) | 410(27.74) | 82.511 | <0..001*** |
| Yes | 5358(78.38) | 3178(59.31) | 2180(40.69) |  |  |
| **Total** | 6836(100) | 4246(62.11) | 2590(37.89) |  |  |
| Note: Chi-square tests were used to compare the differences in physical exercise levels among occupational populations with different characteristics; *=*P*<0.05, **=*P*<0.01, ***=*P*<0.001. | | | | | |

# Figure S1 Comparison of pain distribution in various body parts and overall severity of work-related musculoskeletal disorders (WMSDs) among occupational population by physical exercise level. (a): Pain distribution in hands. (b): Pain distribution in shoulders, neck, or upper back. (c): Pain distribution in waist. (d): Pain distribution in knees. (e): Pain distribution in feet. (f): Quantitative comparison of overall WMSDs severity by physical exercise level.
